# Heat therapy for primary dysmenorrhea: A systematic review and meta-analysis of its effects on pain relief and quality of life

**DOI:** 10.1038/s41598-018-34303-z

**Published:** 2018-11-02

**Authors:** Junyoung Jo, Sun Haeng Lee

**Affiliations:** 1 Department of Korean Obstetrics & Gynecology, Conmaul Hospital of Korean Medicine, 110 Seochojungang-ro, Seocho-gu, 06634 Seoul Republic of Korea; 2 Research Institute of Korean Medicine, College of Korean Medicine, Dongguk University, 123 Dongdae-ro, Gyeongju-si, Gyeongsangbuk-do, 38066 Seoul Republic of Korea; 3 Department of Pediatrics of Korean Medicine, Kyung Hee University Korean Medicine Hospital, Kyung Hee University Medical Center, 23 Kyungheedae-ro, Dongdaemun-gu, 02447 Seoul Republic of Korea

## Abstract

Primary dysmenorrhea, which is menstrual pain without pelvic pathology, is the most common gynecologic condition in women. Heat therapy has been used as a treatment. We assessed the evidence on heat therapy as a treatment for primary dysmenorrhea. We searched 11 databases for studies published through July 2018. All randomized controlled trials (RCTs) that addressed heat therapy for patients with primary dysmenorrhea were included. Data extraction and risk-of-bias assessments were performed by two independent reviewers. Risk of bias was assessed using the Cochrane risk-of-bias tool. Six RCTs met our inclusion criteria. Two RCTs found favorable effects of heat therapy on menstrual pain compared with unheated placebo therapy. Three RCTs found favorable effects of heating pads on menstrual pain compared with analgesic medication (n = 274; SMD −0.72; 95% confidence interval −0.97 to −0.48; *P* < 0.001; two studies). One RCT showed beneficial effects of heat therapy on menstrual pain compared with no treatment (n = 132; MD −4.04 VAS; 95% CI −4.88 to −3.20; P < 0.001). However, these results are based on relatively few trials with small sample sizes. Our review provided suggestive evidence of the effectiveness of heat therapy for primary dysmenorrhea, but rigorous high-quality trials are still needed to provide robust evidence.

## Introduction

Primary dysmenorrhea refers to painful menstrual cramps in the lower abdominal region during menstruation in the absence of any discernible macroscopic pelvic pathology^1^. It frequently involves other symptoms, including sweating, headache, nausea, vomiting, diarrhea, and tremulousness before or during menstration^2^. Its estimated prevalence varies between 45% and 95% of all women of reproductive age^3^. Dysmenorrheic pain is the primary cause of recurrent short-term school or work absenteeism among young women of childbearing age^4^. Women with this condition report that menstruation has an immediate negative impact on their quality of life (QoL), whereas women who do not suffer from this condition do not report such an experience during menstruation^4^. Pelvic pain may also cause anxiety and depression, which can amplify the severity of pain^5–7^. Despite its negative effects and the availability of treatment at minimal cost, few patients with primary dysmenorrhea visit medical clinics, and members of this population are frequently undertreated^8,9^.

Nonsteroidal anti-inflammatory drugs (NSAIDs) are considered the primary treatment for primary dysmenorrhea, but they commonly cause adverse effects (AEs), including indigestion, headaches, and drowsiness^10^. Typically, hormone contraceptives are used only for women who are not planning to become pregnant^9^. Therefore, many women also seek alternative therapies, such as heating pads for cramps, to manage their menstrual discomfort^4,11^. A recent systematic review suggested that heat therapy may be related to pain reduction, although rigorous high-quality trials are still needed before conclusive recommendations can be made^11^. However, as the review did not include several important randomized controlled trials (RCTs), another comprehensive review that focuses on the type and method of various heating modalities is needed.

Superficial heat that ranges from 40–45 °C treats the application site to a depth of about 1 cm. Traditionally, superficial heat has been used in different forms (e.g., hot water bags, towels, or bottles) to ease menstrual pain. Although deep heat, such as shortwave diathermy and microwave diathermy, treats deeper structures at depths of 2–5 cm, deep heat also causes vascular and metabolic changes in deeper tissues and organs^12^. Studies have found that heat is a common (36.5–50%) method for coping with dysmenorrhea^13^. For women with dysmenorrhea, the application of local heat can reduce muscle tension and relax abdominal muscles to reduce pain caused by muscle spasms. Heat can also increase pelvic blood circulation to eliminate local blood and body fluid retention and diminish congestion and swelling, thereby enabling a reduction in pain caused by nerve compression^14^. Therefore, in this review, we investigated current evidence related to the effectiveness of heat therapy as a treatment for primary dysmenorrhea. All RCTs dealing with heat therapy for patients with primary dysmenorrhea were analyzed to compare the effects of this treatment with those of control treatments on pain indicators.

## Results

### Description of included trials

**Figure 1:**
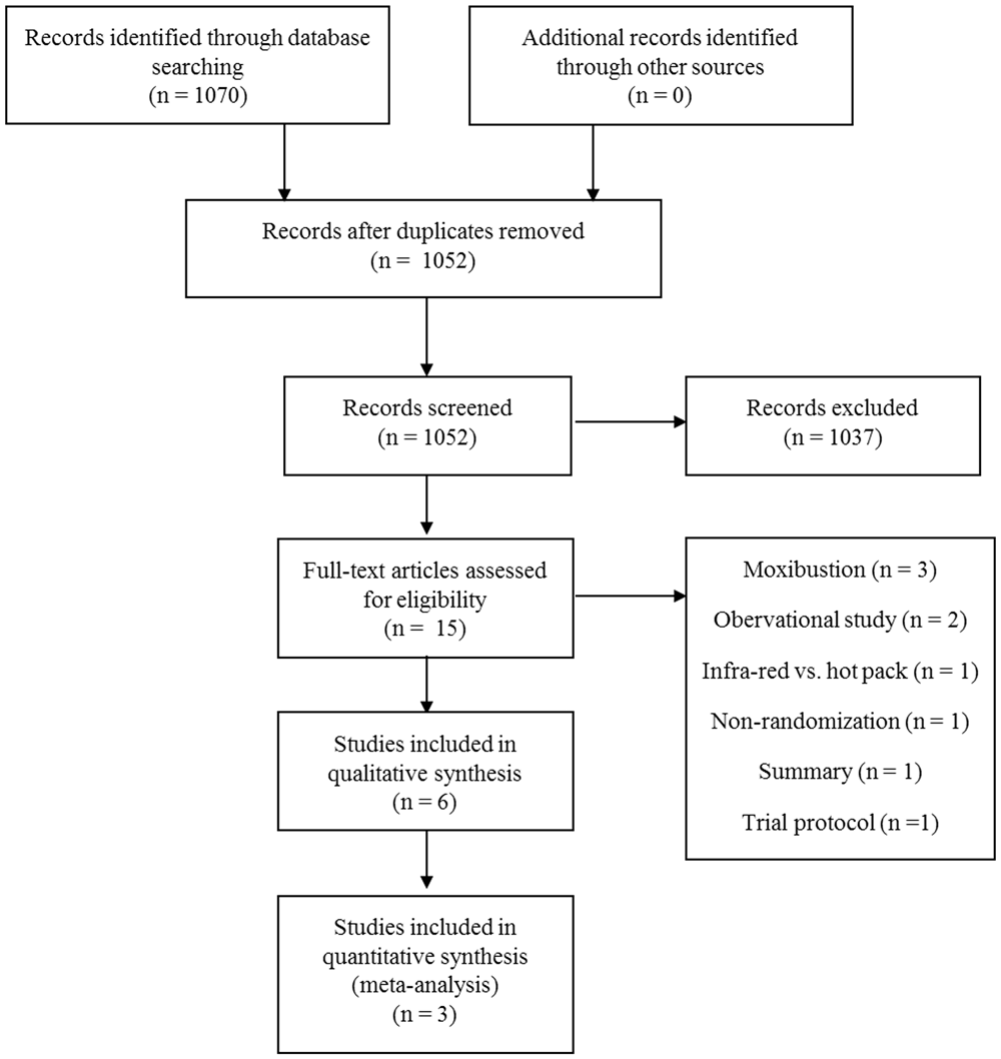
PRISMA chart of the heat therapy.

After removing duplicates, 1052 studies were screened and 15 full-text articles were assessed for eligibility. Three studies that used moxibustion were excluded because it delivers heat and excites the nervous system by acupoint stimulation^15^. Two observational studies, one summary, and one trial protocol were also excluded. One study compared infrared heat to hot packs, and the other study was conducted in a non-randomized setting. Therefore, six RCTs were ultimately included in the analysis (Fig. 1). The characteristics of the included studies are summarized in Table 1. Two RCTs were conducted in America^16,17^, and one RCT each was conducted in Iran^18^, Korea^19^, Taiwan^14^, and Turkey^13^. All of the studies were published in peer-reviewed journals. Four studies used a heating device, such as a patch or wrap^13,16–18^, and two studies used a ceramic belt emitting far-infrared radiation (FIR)^14,19^. Details of the heat treatment are listed in Table 2. Akin *et al*. (2001 and 2004) reported only the mean value^17^ or the mean value and standard error of the reduction in pain scores^16^. Furthermore, the exact number of participants in the intervention and control groups was unclear. Akin *et al*. (2004) reported that 357 participants finished the trial, and 11 participants were excluded; however, they finally analyzed 344 participants^16^. Ke *et al*. just reported pain scores using figures with no numerical values^14^. One of the authors (JJ) contacted the corresponding authors by electronic mail to request additional information, but the authors replied either that they had no raw data^16,17^ or did not respond^14^. Therefore, meta-analyses were performed using the other two studies that compared a heat patch with an analgesic^13,18^. Another study that compared the FIR belt with a heat pack with a placebo belt with a heat pack was reported separately^19^. We used data from the first menstrual cycle after treatment, with the exception of one study, which reported a baseline difference in pain intensity during the first menstrual cycle^13^.

**Table 1:** Baseline characteristics of included studies.

First author	Year	Participant (I/C)	Intervention	Comparison	Pain assessment time	Pain outcome measures	Results(I/C)	Adverse events (I/C)
Akin	2001	20/21	Heated patch + 400 mg/day Ibuprofen	Unheated patch + 400 mg/day Ibuprofen	Day 1: 0, 1, 2, 3, 4, 5, 6, 8, 10, and 12 h Day 2: 0, 2, 4, 6, 8, 10, and 12 h Day 3: 0 hour	A. Pain relief score on 6-point scale B. Reduction in pain intensity during days 1–2 on NRS-101	A. 3.55/3.07 (mean) B. 43.8/39.0 (mean)	None
		20/20	Heated patch + Placebo	Unheated patch + Placebo			A. 3.27/1.95^***^ (mean) B. 40.4/21.9^**^ (mean)	
Akin	2004	151/150	Heat wrap	4000 mg/day Acetaminophen	0.5, 1, 1.5, 2, 3, 4, 5, 6, 7, 8, 24, 48 h	A. Pain relief score during day 1 on 6-point scale B. Abdominal muscle tightness and cramping during day 1 on NRS-101	A. 2.48 ± 1.23/2.17 ± 1.22^*^B. 40.40 ± 20.15/44.50 ± 20.45^*^	Conjunctivitis (1/0) Pink skin (1/0) Headache (0/1) Rhinitis (0/1) Upper respiratory infection (0/1) Anxiety (0/1)
Lee	2011	52/52	Far-infrared belt + Hot water bag	Placebo belt + Hot water bag	Menstrual cycle 3	A. Maximum VAS score B. Participants taking pain medications	A. 5.89 ± 2.16/6.33 ± 2.16 B. 29/28	1^st^-degree burns (3/4) Skin rash (1/0) Itching (4/2) Abdominal discomfort (1/0) Nausea (1/1)
					Menstrual cycle 4		A. 6.13 ± 2.38/5.72 ± 2.38 B. 29/28	
					Menstrual cycle 5		A. 4.96 ± 2.16/5.69 ± 2.16 B. 29/19	
					Menstrual cycle 6^a^		A. 5.04 ± 2.45/5.97 ± 2.45 B. 25/25	
					Menstrual cycle 7^a^		A. 5.08 ± 2.24/6.47 ± 2.24^**^B. 26/25	
Ke	2012	26/25	Far-infrared belt	Placebo belt	1–3 days of menstrual cycle 1–3	Pain score on VRS-6 and NRS-11	General trend towards lower score in far-infrared belt group on 1–3 days of menstrual cycle^**^	Not reported
Navvabi	2012	72/75	Heated patch	400 mg/day Ibuprofen	2, 4, 8, 12, and 24 h after the onset of menstruation	SF-MPQ A. Sensual pain score on 34-point scale B. Emotional pain score on 13-point scale C. Current pain score on 101-point VAS D. Total pain score on 6-point VAS	A. 5.55 ± 6.81/5.55 ± 6.84 B. 2.63 ± 2.60/3.13 ± 2.94 C. 26.54 ± 36.41/26.97 ± 32.91 D. 1.63 ± 1.93/3.57 ± 2.72	Not reported
Potur	2014	66/66	Heated patch	No treatment	Menstrual cycle 1: T1, T2, T3	T2. Mid-treatment pain intensity on 10-cm VAS T3. End of treatment pain intensity on 10-cm VAS	T2. 4.76 ± 2.29/6.58 ± 1.66^b^ T3. 1.99 ± 2.42/5.78 ± 2.63^b^	Not reported
					Menstrual cycle 2: T1, T2, T3		T2. 4.53 ± 2.39/6.90 ± 1.53^b^ T3. 1.90 ± 2.39/5.94 ± 2.51^b^	
		66/61		Self-analgesic drugs	Menstrual cycle 1: T1, T2, T3		T2. 4.76 ± 2.29/5.21 ± 2.60^b^ T3. 1.99 ± 2.42/4.19 ± 3.03^b^	
					Menstrual cycle 2: T1, T2, T3		T2. 4.53 ± 2.39/5.79 ± 2.45^b^ T3. 1.90 ± 2.39/3.61 ± 3.08^b^	

I/C: Intervention/Comparison; ROB: risk of bias; NRS: numerical rating scale; VAS: visual analogue scale; VRS: verbal rating scale; SF-MPQ: shortened revision of the McGill Pain Questionnaire; T1: baseline, T2: after 4 h of intervention (mid-treatment), T3: after 8 h of intervention (end of treatment). Scores are expressed as mean ± standard deviation. *P < 0.05, **P < 0.01. ^a^Post-treatment follow-up period. ^b^There was a significant difference among the three groups at T2 and T3 of the intervention in terms of pain severity (P < 0.001).

**Table 2:** Details of the heat therapies used in the RCTs.

First author	Year	Method	Treatment region	Treatment duration
Akin	2001	Wearing a kidney bean-shaped ultra-thin medical device that supplied heat at a constant temperature of 38.9 °C over a surface area of 180 cm^2^ for 12 hours	Inside the underwear on the lower abdomen	12 h/day for 2 days
Akin	2004	Wearing a heat wrap at a constant temperature of 40 °C	Not reported	8 hours
Lee	2011	A. Wearing a sericite ceramic belt that emitted far-infrared ray (FIR) at a peak wavelength of 5–20 μm when warmed to 40 °C B. 9 × 7 cm disposable hot water bag containing iron powder and other chemicals that quickly heated up to 50 °C and stayed at that temperature for 10 hours	Lower abdomen	While sleeping at night for three consecutive menstrual cycles
Ke	2012	Wearing a 15 × 70 cm belt with 10 wt% FIR ceramic powders including aluminum oxide, ferric oxide, magnesium oxide, and calcium carbonate that emitted 10.16 mW/cm^2^ FIR at a wavelength of 3–16 μm	Abdomen	Entire day during menstruation
Navvabi	2012	Wearing a 7 × 12 cm heated patch	In underwear	Not reported
Potur	2014	Wearing the heat patch containing iron, coal, water, and salt that supplied heat at a constant temperature of 38.9 °C over a surface area of 180 cm^2^ for 8 hours	Lower abdomen	For 8 hours during two menstrual cycles

### Risk of bias in the included studies

**Figure 2:**
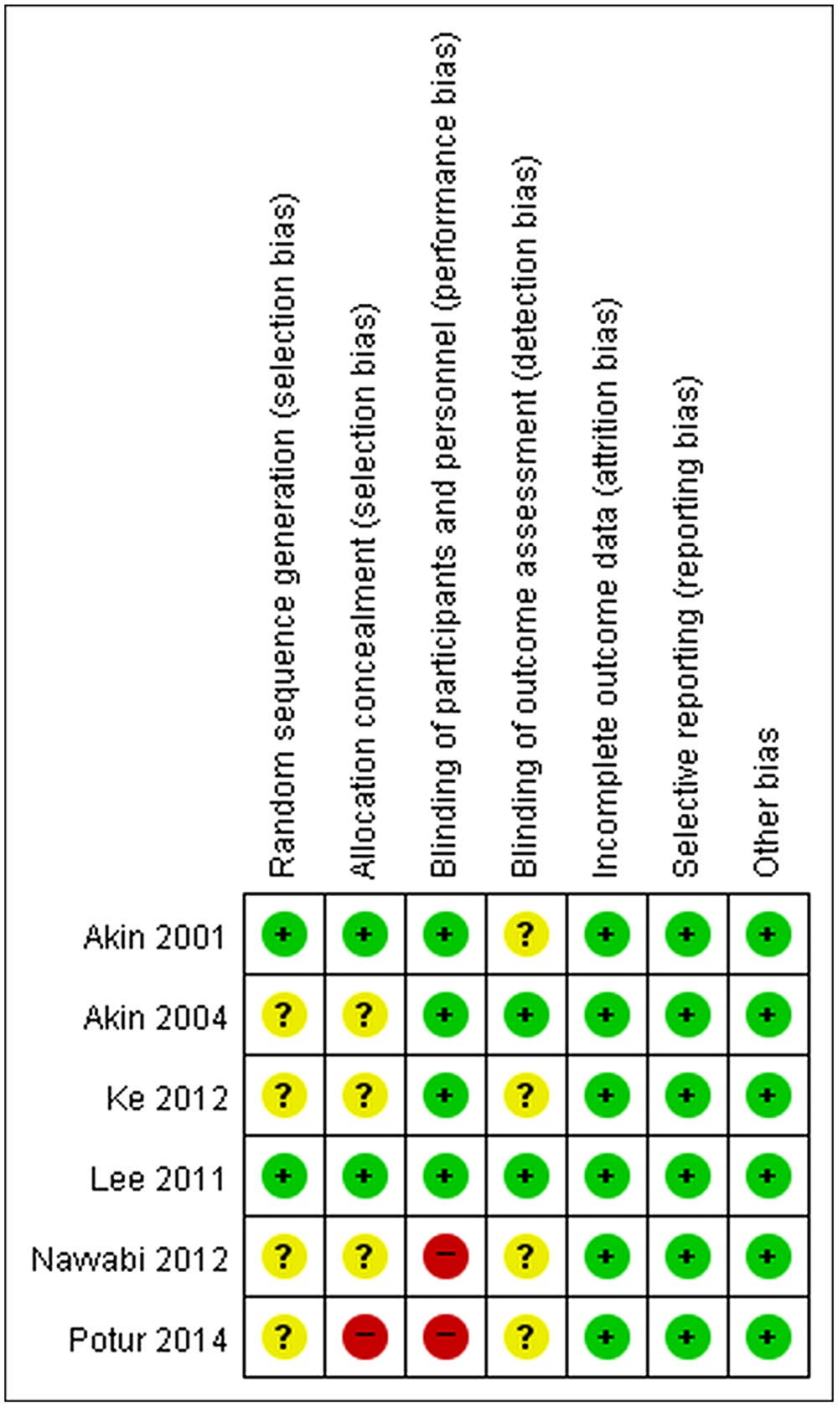
Summary of the risk of bias.

The risk of bias in studies involving random sequence generation and blinding of outcome assessment was low in 33% of the trials (2/6) and unclear in 66% of the trials (4/6). The risk of bias for allocation concealment was low in 33% of the trials (2/6), unclear in 50% of the trials (3/6), and high in 17% of the trials (1/6). The risk of bias in blinding the participants and personnel was high in 33% of the studies (2/6) and low in 66% of the studies (4/6). There was a low risk of bias of incomplete outcome data, selective reporting, and other sources in all studies. Figure 2 summarizes the risks of bias and Appendix S2 provides the authors’ judgments on the risk of bias.

### Outcome measures

#### Self-reported pain severity

**Figure 3:**
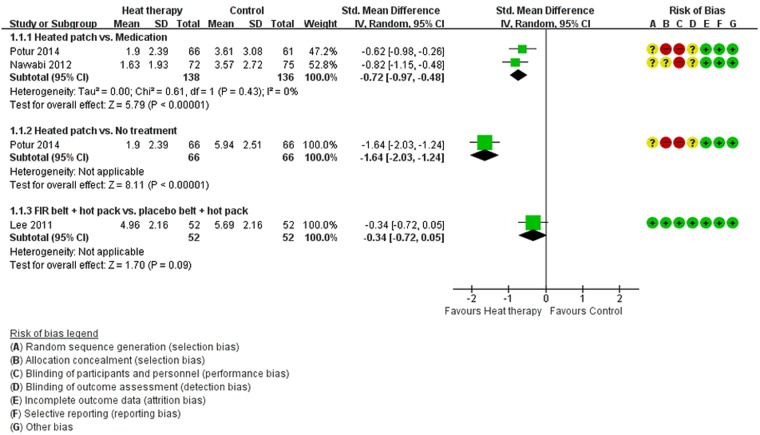
Forest plot of the heat therapy and control.

Each of the six RCTs measured pain severity to identify the effects of heat therapy on alleviating menstrual pain^13,14,16–19^. The meta-analysis of two studies^13,18^ showed that the heat patch had a more favorable effect on the severity of menstrual pain compared with analgesic medication (n = 274; SMD −0.72; 95% confidence interval [CI] −0.97 to −0.48; *P* < 0.001) and no treatment (n = 132; MD −4.04 VAS; 95% CI −4.88 to −3.20; *P* < 0.001) (Fig. 3). In two studies, Akin *et al*. reported that the heat patch demonstrated significant menstrual pain relief compared with unheated placebo therapy^17^ or acetaminophen^16^. However, concurrent use of the heat patch and ibuprofen produced similar pain relief as the combined use of the unheated placebo patch and ibuprofen^17^. Ke *et al*. showed a general trend towards a lower pain score in the FIR-belt group compared with the placebo-belt or blank group^14^. Lee *et al*. found that the FIR belt with a heat pack and the placebo belt with a heat pack had similar effects on pain relief (n = 104; MD −0.73 maximal VAS; 95% CI −1.56 to 0.10; *P* = 0.08) (Fig. 3)^19^. However, we found a significantly greater effect on pain relief in the FIR-belt group (maximal VAS: 5.08 ± 2.24) compared with the placebo-belt group (maximal VAS 6.47 ± 2.24) in the two menstrual cycles immediately following the end of treatment (*P* = 0.002).

#### Validated pain questionnaires

One study used a shortened revision of the McGill questionnaire, which has proven validity and reliability, to measure pain^18^. Pain severity was measured using 11 phrases describing sensory pain (0 to 33 points) and 4 phrases describing emotional pain (0 to 12 points). However, there were no significant differences between the heat patch and ibuprofen in terms of sensual and emotional pain (sensual pain MD = −0.04; 95% CI −2.25 to 2.17; *P* > 0.05; emotional pain MD = −0.50; 95% CI −1.40 to 0.40; *P* > 0.05).

#### Validated quality-of-life questionnaires

One study measured menstrual symptom severity (menstrual quality of life) using a standardized 17-item questionnaire^16^. The four core symptom clusters (pain, negative affect, water retention, and food) were derived from a previous study^20^. The heat-wrap group reported less severe menstrual symptoms (0.91 ± 0.49) compared with the acetaminophen group (0.99 ± 0.49); however, the difference was not statistically significant (MD = −0.08; 95% CI −0.19 to 0.03; *P* = 0.065). The pain cluster score (lower abdominal cramping, low backache, and generalized aches/pains) of the heat-wrap group (1.20 ± 0.74) was significantly lower than that of the acetaminophen group (1.35 ± 0.73; MD = −0.15; 95% CI −0.32 to 0.02; *P* = 0.040). Additionally, the heat-wrap group had significantly lower scores regarding mood swings (*P* = 0.046), fatigue (*P* = 0.012), and lower abdominal cramping (*P* = 0.008).

### Adverse effects

Among the six studies, three noted if AEs were associated with treatment. Specifically, two studies reported AEs^16,19^, and one study had no reported AEs^17^. Mild conjunctivitis and moderate application site reactions occurred in the heat-wrap group, whereas moderate headache, rhinitis, and upper respiratory infection and severe anxiety occurred in the acetaminophen group^16^. The frequency of AEs, including first-degree burns and itching, in the group that used the FIR belt with the heat pack was equal to that of the group that used the placebo belt with the heat pack. All AEs disappeared within a few days without treatment. There were no serious AEs, and no clinically relevant changes in vital signs; no patient discontinued the clinical trial due to an AE^19^.

## Discussion

This systematic review, which included six studies, found that heat therapy appears to decrease menstrual pain in women with primary dysmenorrhea. There was a consistent reduction in menstrual pain with heat therapy compared with unheated placebo therapy. There was also a trend towards a reduction in menstrual pain with heat therapy compared with analgesic drugs. These results appear promising but should be interpreted cautiously because they are based on relatively few trials with an unclear risk of selection bias.

We included only RCTs to remove potential bias and did not have any language restrictions. Although our literature searches included English and Korean databases, and also included searching by hand for relevant articles, we cannot be absolutely certain that all relevant RCTs were found. The meta-analysis included small numbers of studies with relatively small sample sizes. This contributed to imprecision in estimates. There were variations in the duration, type of heat therapy (e.g. patch or wrap or ceramic belt emitting FIR), and duration of follow up used in these studies, leading to heterogeneity in the findings. Akin *et al*. reported only the mean value^17^ or the mean value and standard error of the reduction in pain outcomes^16^. Ke *et al*. reported the outcome only in figures in the paper^14^; therefore, meta-analyses were available from only two studies. A recently published review, which examined the same topic as this article^11^, included a non-RCT that was excluded from our review. Additionally, it failed to include several important studies^16,17,19^ that were included and analyzed in our review.

NSAIDs appear to be an effective treatment for dysmenorrhea, although women using them need to be aware of the substantial risk for AEs^10^. Hormone contraceptives are available only for patients who do not plan to become pregnant^9^. Our systematic review showed the clear benefit of heat therapy for menstrual pain in women with primary dysmenorrhea. Whether this translates into long-term clinical benefits has yet to be demonstrated. One argument for using heat therapy for the management of dysmenorrhea may be that it causes fewer AEs than conventional drugs. However, there was no evidence that there is a difference among them with regard to AEs. If heat therapy were effective and safe for the management of dysmenorrhea in both the short- and long-term, it could become a first-line non-pharmacologic treatment to decrease menstrual pain in women with primary dysmenorrhea, particularly those with contraindications for NSAIDs.

This systematic review and meta-analysis suggests that heat therapy was associated with a decrease in menstrual pain in women with primary dysmenorrhea. These results are consistent with the recommendation of local heat as a complementary treatment for dysmenorrhea^9^. We need to compare the effects of various heating modalities with those of other general interventions in terms of short- and long-term outcomes as well as cost-effectiveness. A well-designed multicenter trial to address this issue and provide robust evidence of benefit is warranted to clarify the role of heat therapy in this population.

## Methods

### Protocol registration

The protocol for this systematic review was registered (CRD42017060127), and the review was conducted and reported as outlined in the Preferred Reporting Items for Systematic Reviews and Meta-Analyses (PRISMA) statement^21^.

### Literature search

We searched the following databases for relevant studies published through July 2018: MEDLINE, EMBASE, the Cochrane Central Register of Controlled Trials, the Allied and Complementary Medicine Database, and the Cumulative Index to Nursing and Allied Health Literature. We also searched six Korean medical databases: the Oriental Medicine Advanced Searching Integrated System, the Korean Traditional Knowledge Portal, the Korean Studies Information Service System, the Research Information Service System, Korea Med, and DBpia. Each search term was composed of a disease term (e.g., dysmenorrhea, menstrual pain, painful menstruation, period pain, painful period, cramps, menstrual disorder, or pelvic pain) and an intervention term (e.g., heat/warm). No language restrictions were imposed. The search strategies are presented in online Supplement 1. Similar search strategies were applied to the other databases. Study selection was documented and summarized in a PRISMA-compliant flow chart (http://www.prisma-statement.org) (Fig. 1)^21^.

### Study selection

#### Types of research

All prospective RCTs, quasi-RCTs, and cluster RCTs were included. Observational, cohort, case–control, and case series studies were excluded as were qualitative, uncontrolled trials, and laboratory studies.

#### Type of participants

Patients of any age with primary dysmenorrhea were included in the systematic review. Dysmenorrhea secondary to other pathologies, such as uterine myoma, endometriosis, or infection, was excluded in this review.

#### Types of intervention

Randomized studies of superficial or deep heat therapy, either as the sole treatment or as an adjunct to other treatments applied in both groups (intervention and control groups) in the same manner, were included.

#### Types of comparisons

We included any type of control intervention, including no treatment, placebo, and conventional medication. RCTs that compared different heat treatments were excluded.

### Outcome measures

#### Primary outcomes

The primary outcomes were reduction of menstrual pain only during the intervention or as a result of the intervention measured using a visual analogue scale (VAS) or numeric rating scale (NRS).

#### Secondary outcomes

The secondary outcomes were scores on validated pain questionnaires, QoL, and AEs.

### Data extraction

Two authors (JJ and SHL) performed the data extraction and quality assessment using a predefined data extraction form. The form included information pertaining to the first author, study design, language of publication, country where the trial was conducted, clinical setting, diagnostic criteria, number of participants allocated to each group, drop-out number, treatment duration, outcome, outcome results, and AEs associated with heat therapy. When studies reported outcomes at more than one time point, a similar measurement point in other studies was used for the analysis, such as at the end of treatment or the first menstrual cycle after treatment. Any disagreement among the authors was resolved by discussion among all authors. When the data were insufficient or ambiguous, JJ contacted the corresponding author by electronic mail or telephone to request additional information or clarification.

### Assessment of risk of bias in the included studies

The risk of bias was assessed using the risk-of-bias assessment tool from the Cochrane Handbook ver. 5.1.0, which includes random sequence generation, allocation concealment, blinding of participants and personnel, blinding of outcome assessments, incomplete outcome data, selective reporting, and other sources of bias^22^. Our review used ‘L’, ‘U’, and ‘H’ to indicate the results of the assessments: ‘L’ indicated a low risk of bias, ‘U’ indicated that the risk of bias was unclear, and ‘H’ indicated a high risk of bias. Disagreements were resolved by discussion among the authors.

### Data synthesis and analysis

Statistical analyses were performed with the program Review Manager (ver. 5.3 Copenhagen: The Nordic Cochrane Centre, The Cochrane Collaboration, 2014). Trials were combined according to the type of intervention and type of outcome measure and/or control. Data were pooled and expressed as the mean difference (MD) or standardized mean difference (SMD) for continuous outcomes using random-effects models, because high levels of heterogeneity had been anticipated.

### Assessment and investigation of heterogeneity

Heterogeneity among studies was assessed using the chi-square (χ^2^) test with a significance level of *P* < 0.1 and the I^2^ statistic^23^. The I^2^ statistic indicates the proportion of variability among trials that was not explained by chance alone, and an I^2^ value > 50% indicates substantial heterogeneity^23,24^. When substantial heterogeneity was detected, we explored the sources of heterogeneity by performing a subgroup analysis according to the type of intervention or control group. If some factors (e.g., lack of included trials, large methodological or clinical differences among trials) were found, we did not conduct a subgroup analysis or data synthesis, but instead created a narrative description of the included studies. We assessed publication bias by using a funnel plot if 10 or more studies were included.

### Missing data

We made our best efforts to analyze data on an intention-to-treat basis, and attempts were made to obtain missing data from the original investigators. When these attempts were unsuccessful, we did not substitute data for missing data but analyzed only the available data.

## Electronic supplementary material

PRISMA checklist

Supplementary information
