# Supplementary material for: Heat therapy for primary dysmenorrhea: A systematic review and meta-analysis of its effects on pain relief and quality of life
Source: Sci Rep. 2018 Nov 2;8:16252. doi: 10.1038/s41598-018-34303-z (PMC6214933; doi:10.1038/s41598-018-34303-z)
Supplement: Supplementary file 2 — Supplementary information [file 41598_2018_34303_MOESM2_ESM.pdf]

Heat therapy for primary dysmenorrhea: A systematic review and meta-analysis of its effects on pain relief and quality of life

Junyoung Jo, Sun Haeng Lee

Supplementary information

Appendix S1. Search strategy

- CENTRAL

1 exp Dysmenorrhea
2 (Dysmenorrh$ or primary dymenorrh$).tw.
3 (menstrual adj5 pain).tw.
4 (painful adj5 mens$).tw.
5 pelvic pain.tw.
6 (menstrual adj5 cramp$).tw.
7 (#1 OR #2 OR #3 OR #4 OR #5 OR #6)
8. (heat$ or hot or warm$).tw.
9. (infrared or infra-red).tw.
10. poultice.tw.
11. (#8 OR #9 OR #10)
12. (#7 AND #11)

- MEDLINE, CINAHL, AMED

1 dysmenorrhea
2 dysmennorrh$
3 primary dymenorrh$
4 menstrual adj5 pain
5 painful adj5 mens$
6 pelvic pain
7 menstrual adj5 cramp$
8 (#1 OR #2 OR #3 OR #4 OR #5 OR #6 OR #7)
9 heat$
10 hot
11 warm$
12 infrared
13 infra-red
14 poultice
15 (#9 OR #10 OR #11 OR #12 OR #13 OR #14)
16 (#8 AND #15)

- Korea MED

1 dysmenorrh*
2 heat*
3 hot

4 warm*
5 infrared
6 poultice
7 (#2 OR #3 OR #4 OR #5 OR #6)
8 (#1 AND #7)

• EMBASE
1 dysmenorrhea
2 pelvic pain
3 pelvic girdle pain
4 (#1 OR #2 OR #3)
5 heat
6 thermotherapy
7 hot water
8 infrared radiation
9 poultice
10 (#5 OR #6 OR #7 OR #8 OR #9)
11 (#4 AND #10)

• Oriental Medicine Advanced Searching Integrated System (OASIS), KTKP
1 Wolgyeongtong
2 Saengritong
3 Wolgyeonggonranjeung
4 (#1 OR #2 OR #3)
5 Onyeol
6 Jeogoeseon
7 Yeol
8 Jjimjil
9 (#5 OR #6 OR #7 OR #8)
10 (#4 AND #9)

Appendix S2. The authors' judgment on risk of bias

Random sequence generation
Two studies reported computerised blocked random sequence generation. The other four studies failed to report the method of random sequence generation.

Allocation concealment
We rated two studies using central allocation concealment as having a low risk of bias. We rated one study as having a high risk of bias because it allocated subjects by a randomly listed rank. The other three studies did not mention any method of allocation concealment.

Blinding of participants and personnel
Four studies using placebo heat therapy were rated as having a low risk of bias. The other two studies were considered to have a high risk of bias because they compared heat therapy with active medication or no treatment.

Blinding of outcome assessment
Two studies with blind investigators were classified as having a low risk of bias, whereas the others were classified as having an unclear risk of bias because they did not describe a blinding procedure for outcome assessment.

Incomplete outcome data
Two studies reported the reasons for incomplete outcome data, including scheduling problems, protocol violations, or drop-outs. Therefore, these were rated as having a low risk of bias. The other studies were rated as having a low risk of bias because they reported no missing data or drop-out rates.

Selective outcome reporting
All studies reported all predefined outcomes; therefore, the risk of bias was low in al l studies.

Table S1. Studies included and excluded and the reasons for inclusion and exclusion.

No.	Included studies	Reason for inclusion
1	Akin MD, Weingand KW, Hengehold DA, Goodale MB, Hinkle RT, Smith RP. Continuous low-level topical heat in the treatment of dysmenorrhea. Obstet Gynecol. 2001;97(3):343–9.	Group 1: Heat patch + ibuprofen<br>Group 2: Unheated patch + ibuprofen<br>Group 3: Heat patch + placebo<br>Group 4: Unheated patch + placebo
2	Akin M, Price W, Rodriguez G Jr, Erasala G, Hurley G, Smith RP. Continuous, low-level, topical heat wrap therapy as compared to acetaminophen for primary dysmenorrhea. J Reprod Med. 2004;49(9):739–45.	Group 1: Heat wrap<br>Group 2: Unheated wrap
3	Lee CH, Roh JW, Lim CY, Hong JH, Lee JK, Min EG. A multicenter, randomized, double-blind, placebo-controlled trial evaluating the efficacy and safety of a far infrared-emitting sericite belt in patients with primary dysmenorrhea. Complement Ther Med. 2011;19(4):187–93.	Group 1: Sericite belt + Hot water bag<br>Group 2: Placebo belt + Hot water bag
4	Ke YM, Ou MC, Ho CK, Lin YS, Liu HY, Chang WA. Effects of somatothermal far-infrared ray on primary dysmenorrhea: a pilot study. Evid-Based Complement Alternat Med. 2012;2012:240314.	Group 1: Far-infrared (FIR) rays<br>Group 2: Non-treatment
5	Navvabi Rigi S, Kermansaravi F, Navidian A, Safabakhsh L, Safarzadeh A, Khazaian S, Shafie S, Salehian T. Comparing the analgesic effect of heat patch containing iron chip and ibuprofen for primary dysmenorrhea: a randomized controlled trial. BMC Women's Health. 2012;12:25.	Group 1: Heat patch<br>Group 2: Ibuprofen
6	Potur DC, Kömürcü N. The effects of local low-dose heat application on dysmenorrhea. J Pediatr Adolesc Gynecol. 2014;27(4):216-21.	Group 1: Heat patch<br>Group 2: Self-medication<br>Group 3: Non-treatment
No	Excluded studies	Reason for exclusion
1	Kessenich CR. Continuous topical heat was as effective as ibuprofen for dysmenorrhea. Evid-Based Nurs. 2001;4:113.	Summary of Akin MD, Weingand KW, Hengehold DA, et al. Continuous low-level topical heat in the treatment of dysmenorrhea. Obstet Gynecol 2001;97:343–9
2	Kim SO, Cho SH. The Effect of hand acupuncture therapy and moxibustion heat therapy on dysmenorrhea women. Korean J Women Health Nurs. 2001;7(4):610-21.	Moxibustion study
3	Hong YR. [Effects of heat therapy using a far infrared-ray heating element for dysmenorrhea in high school girls]. J Korean Acad Nurs. 2011;41(1):141-8.	Non-randomized study
4	Bharthi HP, Murthy SN, Babina N, Kadam A, Rao MR. Management of pelvic pain in primary dysmenorrhea using a hot hip-bath: a pilot study. Alternat Ther Health Med. 2012;18(1):24–5.	Observational study
5	Song Y, Xu F, Yang Y, Tang X. The therapeutic effect between heat sensitization and non-heat-sensitization after moxibustion at RN4 on patients of primary dysmenorrhea: A clinical study. Int J Clin Acupunct. 2012;21(2):42.	Moxibustion study
6	Chaudhuri A, Singh A, Dhaliwal L. A randomised controlled trial of exercise and hot water bottle in the management of dysmenorrhoea in school girls of Chandigarh, India. Indian J Physiol Pharmacol. 2013;57(2):114–22.	Observational study
7	Gao J, Wang Q, Xian S, Feng YM, Cao WX, Ye JY, Zhang QQ, Zou CL, Wu QG, Liu SJ. The effect of moxibustion on alleviating menstrual pain in a population of young nursing students: A prospective randomized cross-over pilot study. Complement Ther Med. 2015;23(6):773–81.	Moxibustion study
8	Perez Machado AF, Perracini MR, Cruz Saraiva de Morais AD, da Silva BO, Driusso P, Liebano RE. Microwave diathermy and transcutaneous electrical nerve stimulation effects in primary dysmenorrhea: clinical trial protocol. Pain Manag. 2017;7:359-66.	Study protocol
9	Lee J, Kim K. Effect of near-infrared rays on female menstrual pain in Korea. Nurs Health Sci. 2017;19:366-72.	Infrared vs. Hot packs
